# Fusion of a maxillary third molar with a supernumerary fourth molar: A case report

**DOI:** 10.1002/ccr3.8484

**Published:** 2024-02-06

**Authors:** Ioulianos Apessos, Ioannis Memis, Georgios Mikrogeorgis, Antigoni Delantoni, Dimitrios Dionysopoulos, Theodoros Lillis

**Affiliations:** ^1^ Department of Dentoalveolar Surgery, Implantology and Oral Radiology, School of Dentistry, Faculty of Health Sciences Aristotle University of Thessaloniki Thessaloniki Greece; ^2^ Division of Dentistry 424 General Military Training Hospital Thessaloniki Greece; ^3^ Department of Operative Dentistry, School of Dentistry, Faculty of Health Sciences Aristotle University of Thessaloniki Thessaloniki Greece; ^4^ Department of Endodontology, School of Dentistry, Faculty of Health Sciences Aristotle University of Thessaloniki Thessaloniki Greece

**Keywords:** double tooth, case report, dental anatomy, dental photography, fused teeth, supernumerary tooth

## Abstract

**Key Clinical Message:**

Dental fusion should be included in differential diagnosis when panoramic radiograph reveals changes in tooth shape or size. The use of specialized dental photographic techniques can augment the dentists' knowledge and awareness of such conditions.

**Abstract:**

Dental fusion of impacted teeth may show up as a change in tooth shape and size on the first radiographic examination. This report presents an impacted maxillary third molar fused with a peg‐like distomolar in a 20‐year‐old male. The patient presented with symptoms of localized periodontitis distal to the second molar, and radiographic examination revealed abnormal dental structure of the third molar. Surgical extraction of the impacted molar was the treatment of choice. The final diagnosis of fusion was based on data from ex vivo CBCT, photographs of the extracted tooth, and extracted tooth's sections using polarizing filters. Two‐dimensional radiographs may conceal special dental anatomies. Visualization of such cases using CBCT and dental photography serves to educate dentists and reduce postoperative complications. Knowledge and awareness of possible dental abnormalities are of utmost importance for successful treatment planning.

## Graphical abstract

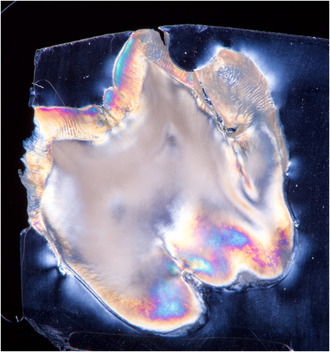

## 1 CASE IMAGE

**FIGURE 1:**
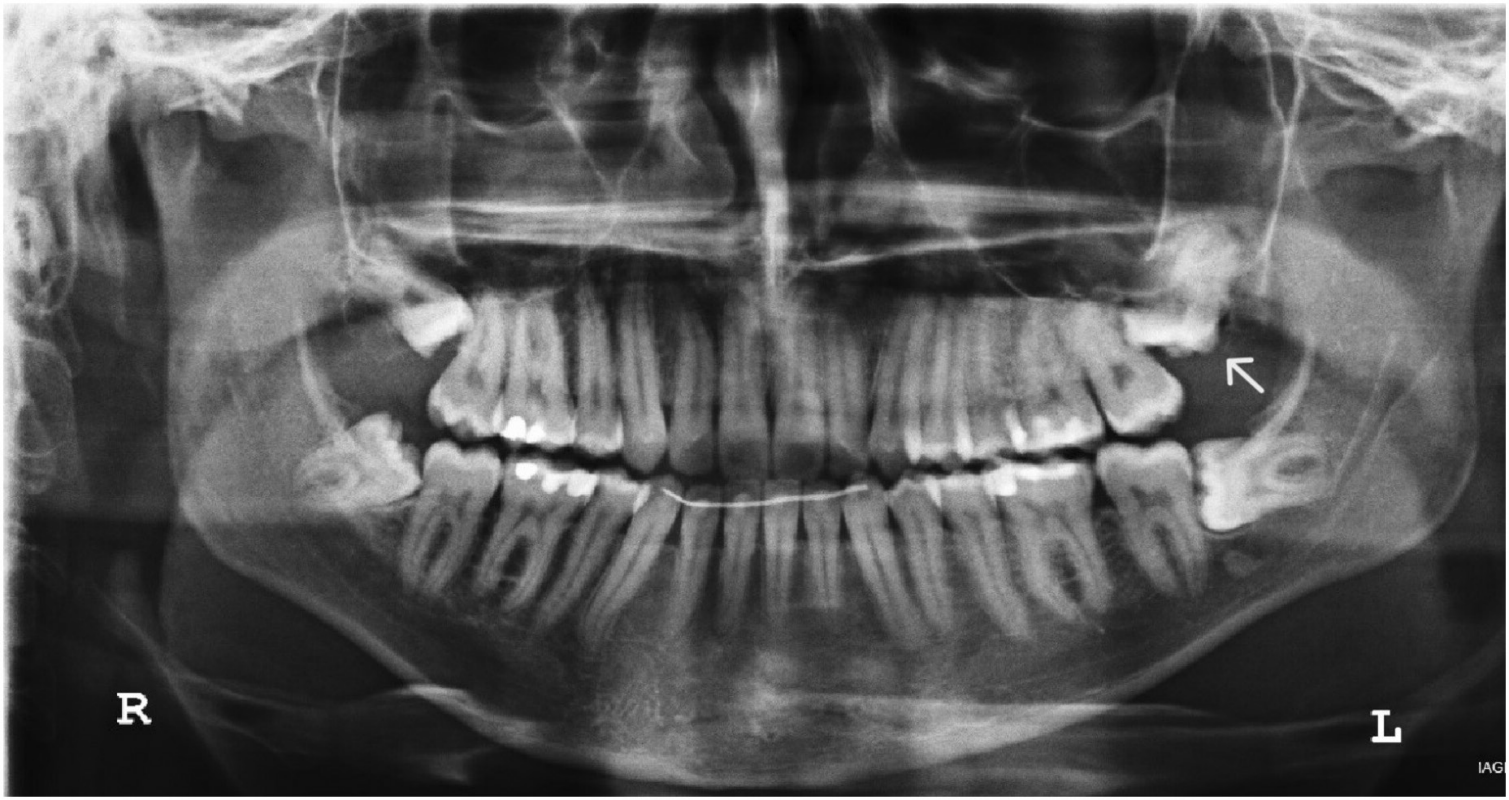
Initial panoramic radiograph. White arrow shows the impacted third molar with small changes in size and shape.

A 20‐year‐old Caucasian male presented to the Department of Dentoalveolar Surgery at the School of Dentistry in February 2023 for intermittent pain in the left side of the maxilla and face. Intraoral examination revealed probing pocket depth distal to the left maxillary second molar 7 mm and bleeding on probing. The medical history revealed no health problems. The panoramic radiograph showed an impacted maxillary third molar that was angled mesially, had a moderate depth of impaction, was abnormal in shape and size, and was in close proximity to the floor of the maxillary sinus (Figure 1). The final diagnosis was localized periodontal disease due to an impacted third molar. Retention of an impacted third molar can compromise the bone that houses the second molar and predispose it to periodontal defects. This situation may ultimately lead to root caries and/or mobility of the second molar. Thus, surgical extraction of the impacted maxillary third molar was the treatment of choice.

**FIGURE 2:**
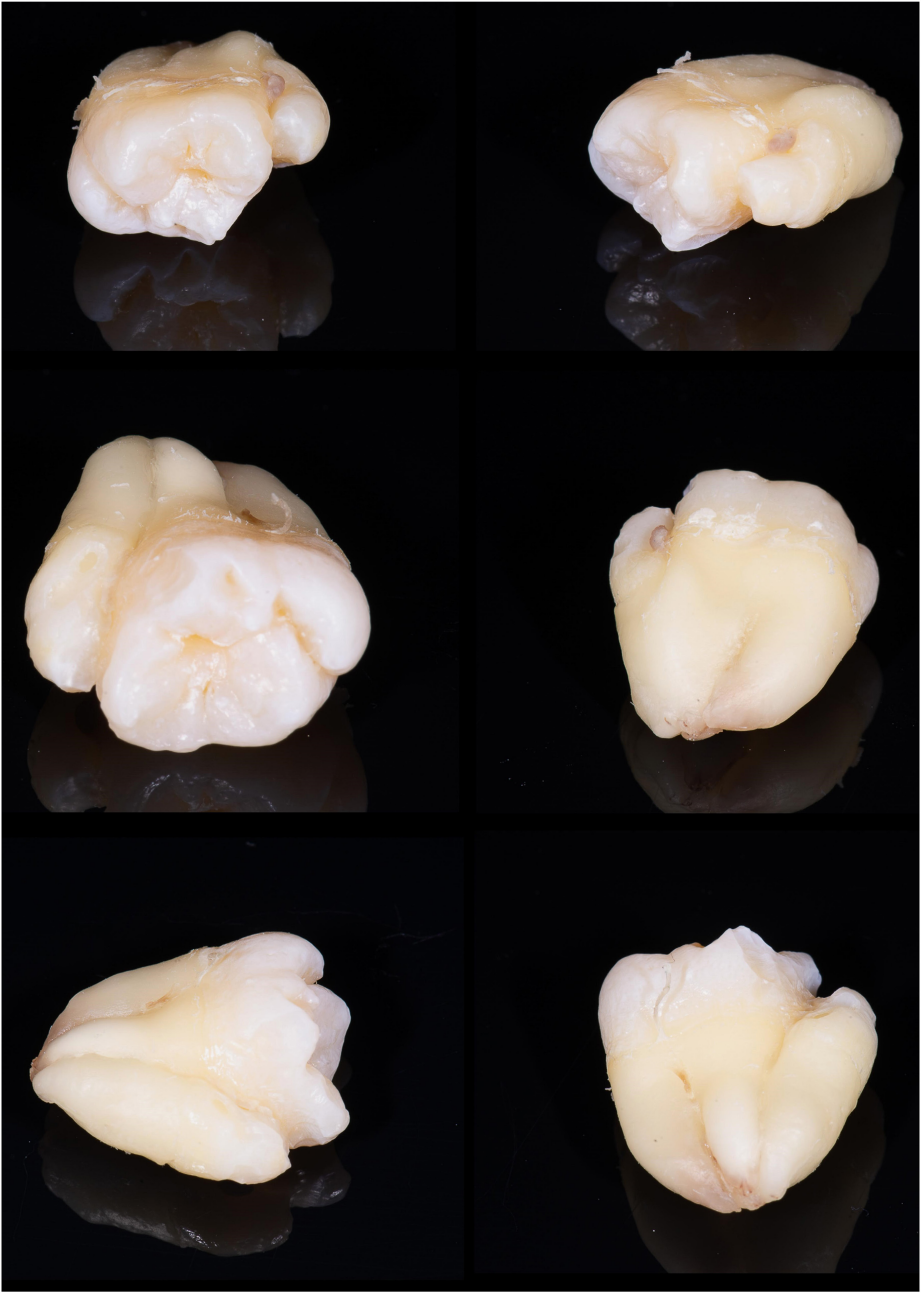
Images of the extracted double tooth from different aspects.

**FIGURE 3:**
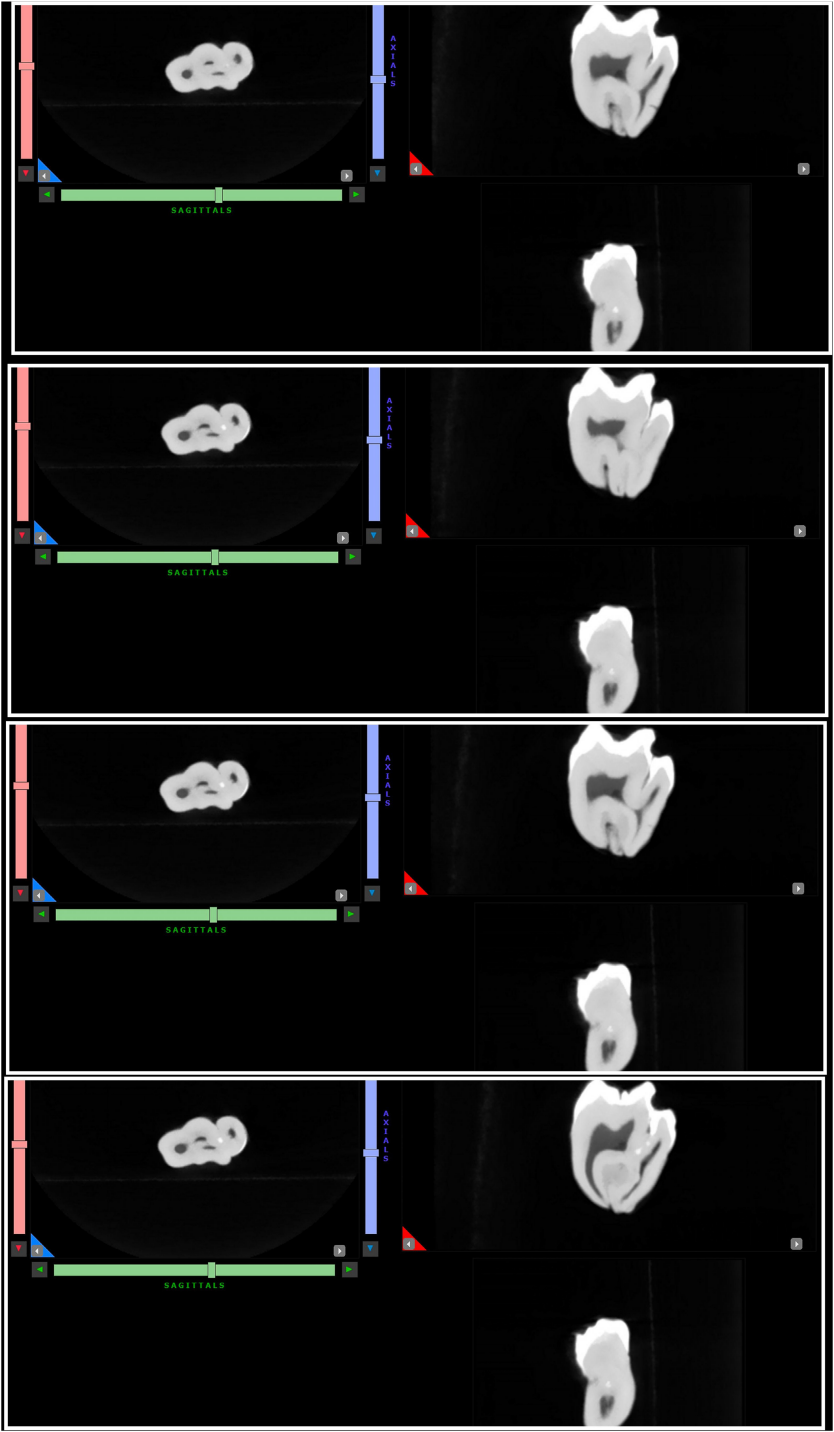
Images exported from ex vivo CBCT of extracted double tooth. Shared pulp canal system is noticed.

**FIGURE 4:**
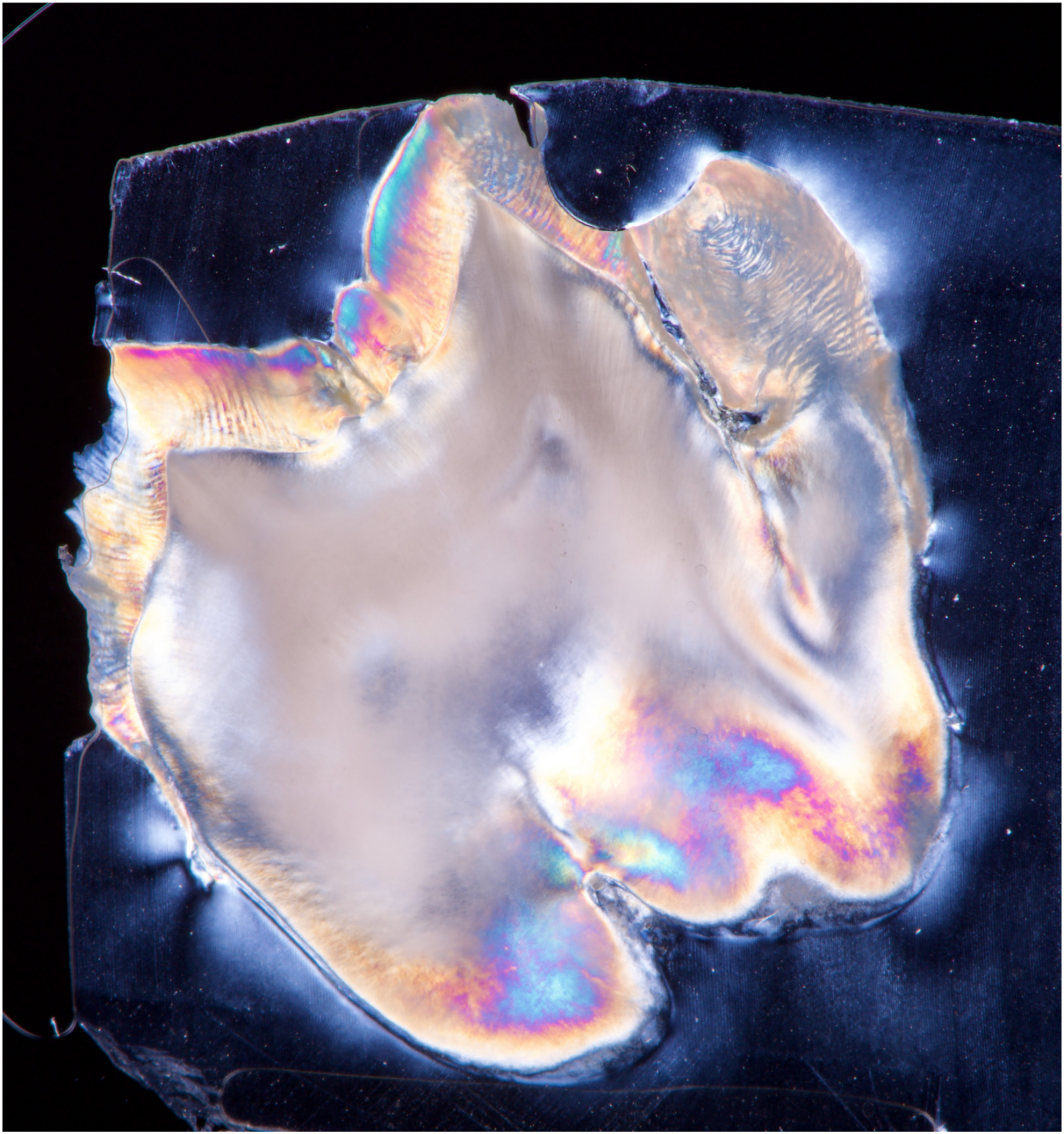
Image of the first tooth section, using polarizing filters, DSLR, macro lens, and flash.

**FIGURE 5:**
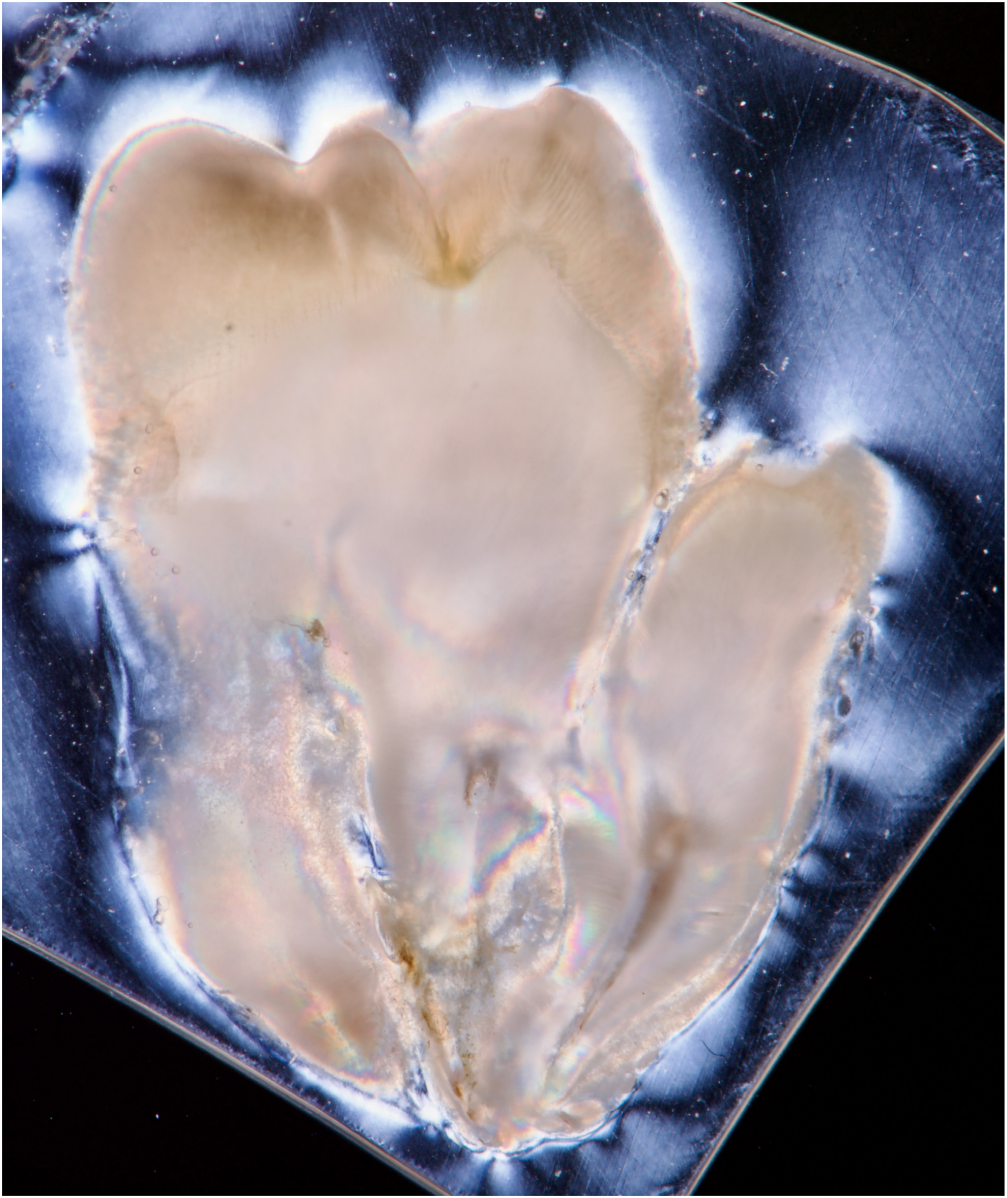
Image of the second tooth section, using polarizing filters, DSLR, macro lens, and flash.

**FIGURE 6:**
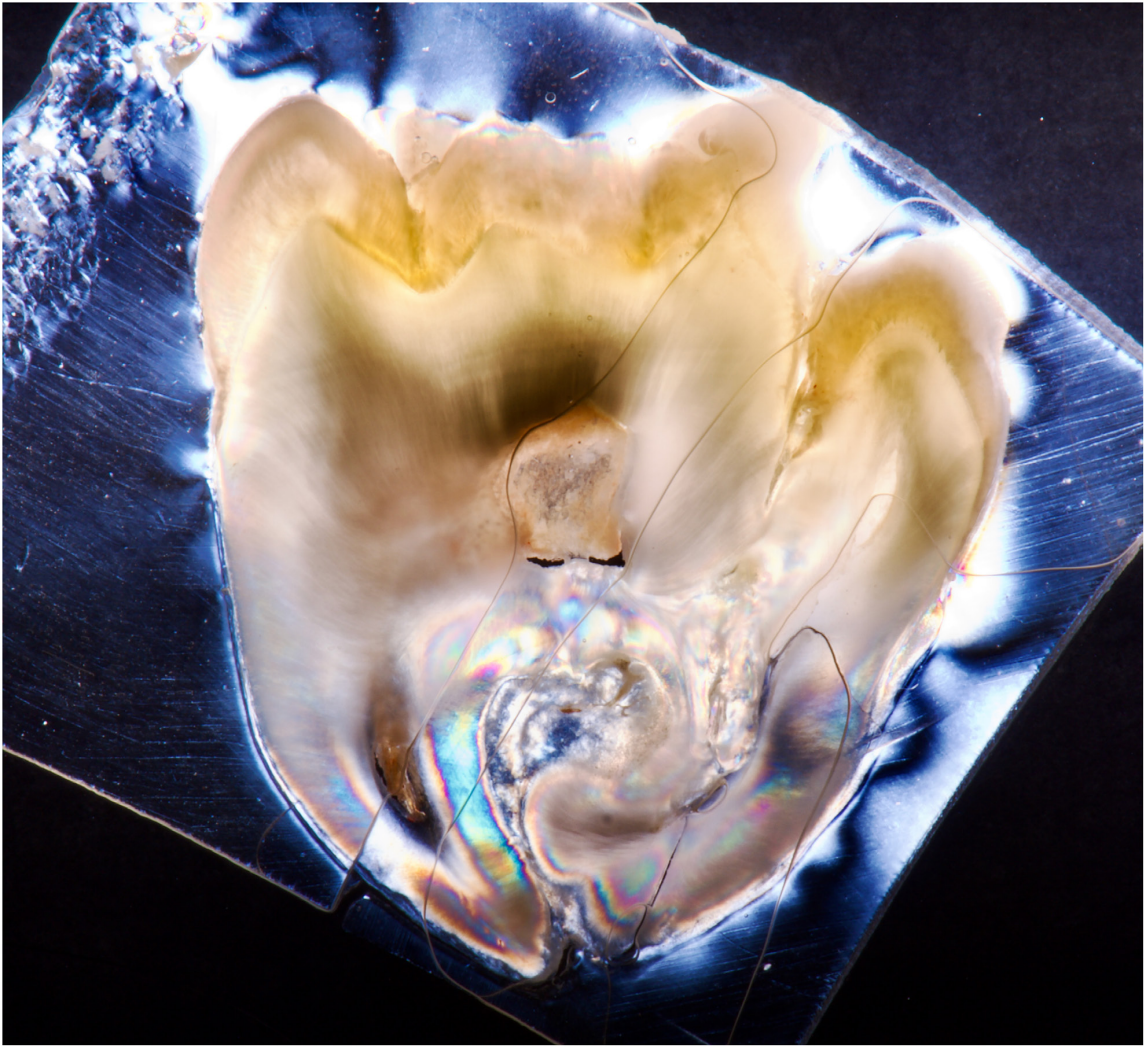
Image of the third tooth section, using polarizing filters, DSLR, macro lens, and flash.

Preoperatively, 2 g of amoxicillin was prescribed. Surgical extraction was performed under local anesthesia. Infiltration anesthesia with lidocaine 2% with 1:80,000 epinephrine was administered. A full‐thickness mucoperiosteal flap was prepared and raised, and then an ostectomy was performed using a surgical handpiece and sterile saline irrigation. Tooth extraction was performed with straight and Warwick‐James elevators. The Valsalva maneuver was negative. The extraction socket was irrigated with saline, and the flap was repositioned and sutured. Niflumic acid 250 mg was prescribed for 3–5 days. The sutures were removed after 1 week and healing was unproblematic. Oral examination after 6 months showed complete healing of the soft tissues. Ex vivo examination of the tooth confirmed the diagnosis of a double tooth. To obtain as much information about tooth anatomy, photographs were taken of all aspects of the tooth, and ex vivo CBCT examination of the tooth was performed (Figures 2 and 3). In addition, the tooth was immersed in epoxy resin. After setting, three sections of the tooth were cut using a low‐speed precision cutting machine (Isomet 11–1180 Buehler, Lake Bluff, IL, USA) with water cooling. The cut surface of each tooth sample was ground on a polishing machine (Jean Wirtz TG 250, Düsseldorf, Germany) at 200 rpm under water cooling (50 mL/min) using 600‐, 800‐, and 1000‐grit silicon carbide abrasive papers (Apex S System, Buehler, Lake Bluff, IL, USA) for 20 s each. The final tooth sections were <1 mm. The tooth sample was placed between two linear polarizing filters. Then the flash (Speedlight SB‐700, Nikon, Japan) with softbox (Godox, China) was held from one side and the DSLR camera (D7200, Nikon, Japan) with macro lens (Micro Nikkor 105 mm, Nikon, Japan) was held from the other side. The filters were crossed in different directions until the desired result was achieved (Figures 4, 5, 6).

Fused teeth are associated with syndromes such as osteopetrosis, chondroectodermal dysplasia, and achondroplasia, and can be caused by environmental factors such as the fetal alcohol exposure, hypervitaminosis or thalidomide use during pregnancy.^1^ The prevalence varies between 0.3% and 3.8% and is higher in the anterior region, in the primary dentition, and males.^2^ Impacted fused molars are rare and are more common in the maxilla than the mandible, while the fourth molar is the most common supernumerary molar.^2^ Although these conditions are asymptomatic in some cases, complications such as dental caries, malocclusion, misalignment, arch asymmetry, and functional problems can occur.^3^ Even if no treatment is planned, the patient should be adequately informed and counseled for dentolegal issues to be avoided.^4^

The differential diagnosis between the different subcategories of double tooth is difficult. The assumption that gemination has a single root canal and fusion has multiple root canals is controversial. The fusion of a normal tooth with supernumeraries still results in a normal tooth count.^5^ Given the features suggestive of both diagnoses, this case indeed presented a diagnostic dilemma.

The fusion of two impacted teeth obviously results in a larger dental structure, which makes the extraction more invasive and inevitably increases the possibility of complications. The close proximity of the maxillary third molar with the maxillary sinus floor should be appreciated. In cases with extensive ostectomy, fracture of the maxillary tuberosity may occur.

## 2 CONCLUSIONS

In summary, changes in tooth size and shape on the initial radiographic examination may be the first sign of dental anomalies. The use of modern imaging techniques, including CBCT and dental photography, may highlight such specific dental anatomies and complement dentists' training in this field. Raising dentists' awareness will lead to careful treatment planning and ensure a successful outcome.

## AUTHOR CONTRIBUTIONS

**Ioulianos Apessos:** Conceptualization; data curation; formal analysis; funding acquisition; investigation; methodology; project administration; resources; software; validation; visualization; writing – original draft; writing – review and editing. **Ioannis Memis:** Investigation; methodology; software; visualization. **Georgios Mikrogeorgis:** Methodology; resources; visualization; writing – review and editing. **Antigoni Delantoni:** Resources; software. **Dimitrios Dionysopoulos:** Supervision. **Theodoros Lillis:** Data curation; investigation; methodology; supervision; visualization; writing – review and editing.

## FUNDING INFORMATION

The authors report no funding for this article.

## CONFLICT OF INTEREST STATEMENT

All authors declare no conflict of interest.

## ETHICS STATEMENT

The authors declare that the individual, whose data were reported in this article, has given written consent to the authors.

## CONSENT

Written informed consent was obtained from the patient to publish this report in accordance with the journal's patient consent policy.

## Data Availability

All data are available upon request.
